# A seeding based cellular assay of tauopathy

**DOI:** 10.1186/s13024-016-0100-9

**Published:** 2016-04-26

**Authors:** Yin Xu, Heidi Martini-Stoica, Hui Zheng

**Affiliations:** Huffington Center on Aging, Baylor College of Medicine, Houston, TX USA; Interdepartmental Program of Translational Biology and Molecular Medicine, Baylor College of Medicine, Houston, TX USA; Medical Scientist Training Program, Baylor College of Medicine, Houston, TX USA; Department of Molecular and Human Genetics, Baylor College of Medicine, Houston, TX USA

**Keywords:** Tauopathy, Prion hypothesis, Neurofibrillary tangles, Cellular model

## Abstract

**Background:**

Tauopathy is characterized by neurofibrillary tangles composed of insoluble hyperphosphorylated tau protein. Currently, cellular models that mimic neurofibrillary tangles in vitro are lacking. Previous studies indicate that neurofibrillary tangles form via a prion replication mechanism. In the present work, we establish a seeding based cellular model according to the prion hypothesis.

**Results:**

We show that cellular soluble tau can be converted to insoluble tau by seeds from the brain lysate of rTg4510 mice or synthetically generated preformed tau fibrils (PFFs). The cellular insoluble tau exhibits classic features of neurofibrillary tangles. Using genetic and pharmacological methods, we demonstrate that inhibition of autophagy increases whereas enhancement of autophagy reduces insoluble tau in our seeding based cellular model. The insoluble tau can be detected and quantified by thioflavin-S staining, thus allowing us to adapt our cellular model to a high-content image-based screening platform.

**Conclusions:**

Our seeding based cellular model reproduces neurofibrillary tangle pathology in vitro and serves as a useful tool for studying tauopathy and identifying tau modulators.

## Background

Tauopathy is a group of neurodegenerative diseases characterized by the accumulation of neurofibrillary tangles (NFTs) composed of tau protein aggregates within the neurons [1]. The most well-known tauopathy is Alzheimer’s disease in which NFTs develop and progress along defined network connections and form the basis for the widely accepted pathologic staging of AD, the Braak Staging [2]. Compelling evidence supports the notion that neurofibrillary tangle pathology causes neurodegeneration in and of itself [3, 4]. Besides AD, NFTs are observed in other tauopathies as well, such as frontotemporal dementia, progressive supranuclear palsy, and Pick’s disease among others [1]. Recently, Huntington’s disease has also been described as a type of tauopathy [5–7].

In physiological conditions, tau protein is unstructured and associates with microtubules in neuronal axons. However, under pathological conditions, tau protein is hyperphosphorylated, which leads to its dissociation from the microtubules and formation of paired helical filaments, referred to as neurofibrillary tangles, in cell bodies and dendrites [1]. Due to its strong hydrophilic nature, the tau protein typically does not form a well-ordered fibrilar structure in vitro. Two methods have been used to produce tau fibrils. One is through charge compensation by polyanions to form tau filaments from recombinant tau protein. The other is through cellular expression of truncated tau that displays high amyloidogenic potential [3]. Different truncations of tau have been used by different laboratories to overcome the lack of amyloidogenic propensity and cytotoxicity of the full length tau. The repeat domain (RD) of tau that binds to microtubules makes up the most commonly used version of truncated tau [8]. Besides the RD, C-terminal truncated tau has been shown to be degraded through the autophagy lysosomal degradation pathway [5], N-terminal truncated tau changes the ability to bind and stabilize microtubules [9], and the calpain-cleaved 17kD tau fragment exhibits prominent neuronal toxicity [10, 11]. Although these cellular models are useful to study tau aggregation, there are a number of disadvantages. First, it is rare to produce insoluble tau in these cells even using truncated tau. Among those models, thioflavin-S positive staining is only observed in tau RD Δ280 cell models and the efficiency is very low (about 6 % of cells are thioflavin-S positive) [12]. Second, truncations of tau may change the intrinsic features of full length tau. Third, there is still no solid evidence to support the existence of truncated tau in animal models or in human brains. Last, truncated tau may induce strong cytotoxicity, which makes maintaining a stable cell line difficult [13]. The aforementioned concerns could vastly explain the difficulties in translating results from current truncated tau cellular models to animal studies.

Although the precise mechanism of neurofibrillary tangle formation is not fully understood, several studies have implicated that it is likely subject to prion-like pathogenesis [13, 14]. The prion protein can exist in different conformations. Under the pathological conformation, monomeric prion protein can form small oligomeric seeds at a very slow rate. After seed formation, monomeric prion protein attaches to the two terminal ends of the seed and forms amyloid filaments at a much faster rate [15, 16]. This hypothesis could explain the challenges in forming NFTs in cells as compared to mouse models. Since cellular models can only be maintained for a matter of days or weeks, it may not be sufficient to allow seed formation. In contrast, the NFTs pathology can develop over a long period of time in mouse or human brains. Here, we seek to establish a cellular assay of neurofibrillary tangles using full length tau to avoid the shortcomings of the truncated tau. Building on the prion hypothesis, we used brain lysate from rTg4510 mice or PFFs as seeds to bypass the seed formation step in cells. As a result, we successfully accelerated cellular NFTs formation and established a seeding based cell assay. Furthermore, we validated this assay by manipulating the autophagy-lysosomal pathway molecules. Finally, using thioflavin S staining as a detection method, we adapted this assay to a high-content capacity to allow screening of insoluble tau modulators.

## Results and discussion

### Establishment of a seeding based cell assay for tauopathy

**Fig. 1:**
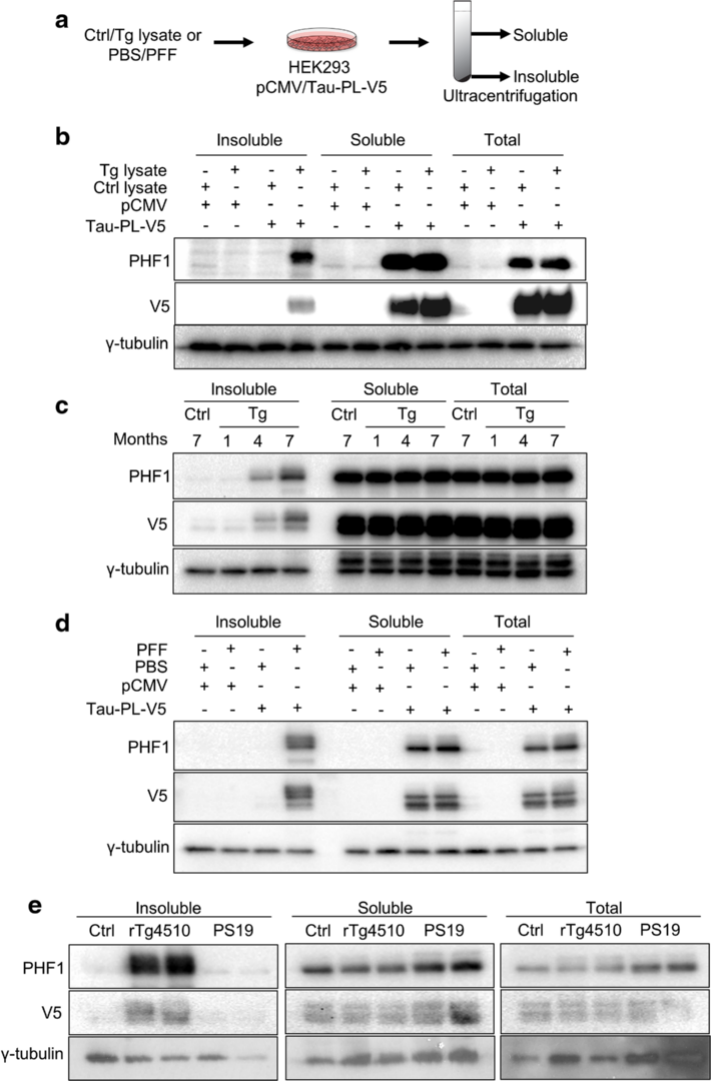
Establishment of seeding based cell assay for tauopathy. **a** Experimental scheme for the seeding based cellular assay. Brain lysate from rTg4510 mice or PFFs were added into the medium of HEK293 cells expressing Tau-PL-V5 (1:1000 dilution for brain lysate and 1:250 dilution for PFF). After 1 day incubation, cell lysate was subjected to ultracentrifugation to isolate soluble and insoluble protein pool. **b** Cells were transfected with Tau-PL-V5 plasmid. pCMV5 plasmid was used as control. The transfected cells were then treated with Tg or control brain lysates. Western blots show that cellular insoluble tau could only be induced in cells expressing Tau-PL-V5 and treated with Tg brain lysate, but not in other groups. **c** Cells were transfected with Tau-PL-V5 plasmid. pCMV5 plasmid was used as control. Transfected cells were treated with equal amount of the brain lysates from 1, 4 and 7 month old Tg mice. Western blots show that seeding activity of brain lysate increased in an age dependent manner. **d** Cells were transfected with Tau-PL-V5 plasmid. pCMV5 plasmid was used as control. The transfected cells were treated with PFF or PBS. Western blots show that PFFs also could be used as seeds to trigger insoluble tau in seeding based cellular assay. **e** Cells were transfected with Tau-PL-V5 plasmid. The transfected cells were treated with control, 4 month rTg4510 or 9 month PS19 brain lysates. Western blots show that only rTg4510 brain lysate could trigger insoluble tau in seeding based cellular assay

Recent studies have shown that pathological tau exhibits prion-like replication and spreading phenomenon induced by seeding [13, 14]. Therefore, we designed a seeding based cellular tauopathy model (Fig. 1a and described in detail in Methods). Because prion amyloid amplification occurs in a sequence-dependent manner, brain lysate from aged rTg4510 tau transgenic mice was used as seeds and added into the culture medium of HEK293 cells expressing vector or tau-PL-V5 containing the same tau sequence (Fig. 1b). In HEK293 cells transfected with the pCMV control plasmid, treatment with brain lysate from control (Ctrl lysate) or tau transgenic (Tg lysate) mice failed to induce insoluble tau (Fig. 1b). This is also the case when control brain lysate (Ctrl lysate) was applied to cells transfected with tau-PL-V5 plasmid (Tau-PL-V5). In contrast, insoluble tau was induced only in tau-PL-V5 transfected cells treated with brain lysate from rTg4510 transgenic mice (Fig. 1b, Tg lysate +; Tau-PL-V5 +). Insoluble tau was V5 positive, demonstrating that it is converted from cellular soluble tau, not from residual brain lysate. Taking into consideration that the intensity of insoluble PHF1 bands is about half of the soluble PHF1 (Fig. 1b) and that approximately 5-fold more insoluble proteins than soluble proteins were loaded, we estimated that the conversion rate is roughly 1/10 (insoluble/soluble), consistent with low conversion efficiency reported in previous studies [17]. It is known that pathological tau accumulates with aging in the brain of rTg4510 mice. To further confirm that insoluble tau is induced by seeding, we used brain lysate from different ages of the mice as seeds (Fig. 1c). As expected, the amount of insoluble tau (both PHF1 and V5 positive) was increased in cells treated with older Tg brain lysate compared to cells treated with younger lysate, suggesting that the seeding activity of Tg brain lysate increases in an age dependent manner. Besides the brain lysate, synthetic tau fibrils have also been used as seeds to induce tau pathology [13, 14]. We thus used PFFs prepared from recombinant synthetic K18-PL that contains the microtubule-binding domain of tau with the same P301L mutation as seeds. As shown in Fig. 1d, K18-PL PFF also induced insoluble tau in tau-PL-V5 transfected cells similar to the brain lysate. In our study, neither the rTg4510 brain lysates nor K18-PL PFF was able to convert the wild type tau to insoluble form (data not shown). Further, application of the same amount of insoluble seeds as that of rTg4510 from another tau transgenic mice, PS19, which expresses the P301S instead of P301L mutation, failed to convert the tau-PL-V5 to insoluble form at any appreciable levels (Fig. 1e, compare rTg4510 vs. PS19, Insoluble vs. Soluble), suggesting that either the conversion requires the same host-agent sequence or that the conversion efficiency induced by different mutations is too low to be detected in our assay.

### Characterization of seeding induced tau pathology

**Fig. 2:**
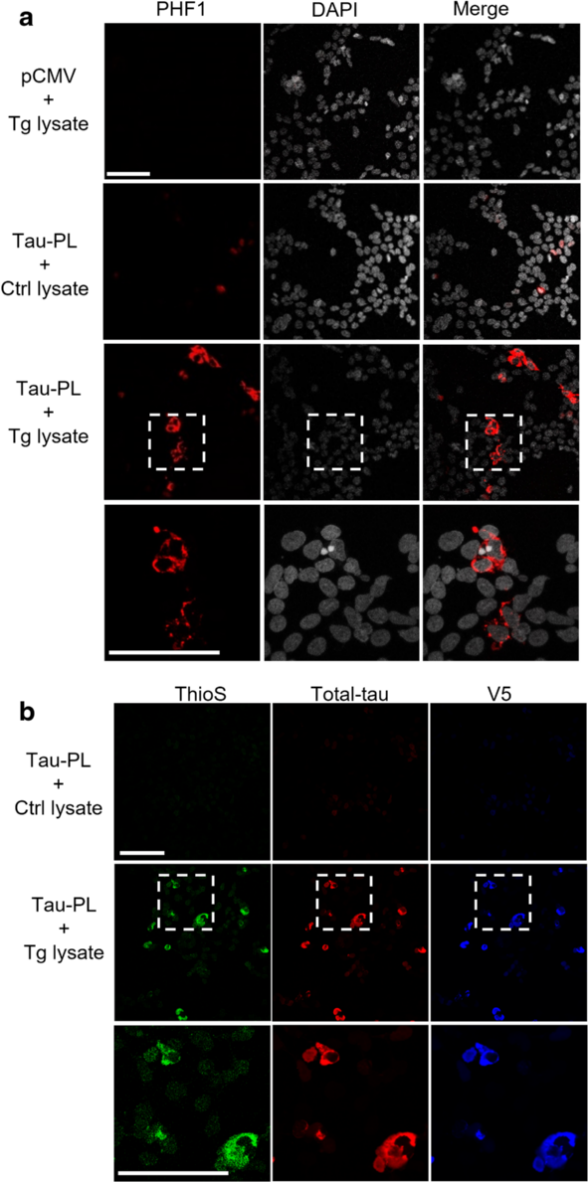
Characterization of seeding induced tau. **a** Soluble proteins were extracted by 1 % Triton X-100 during fixation. Representative images show that insoluble tau is recognized by phosphorylated tau antibody PHF1 in cells expressing Tau-PL-V5 and treated with Tg brain lysate, but not in other groups. The higher magnification views were shown in the bottom panel. **b** Representative images show that insoluble tau is thioflavin-S, total-tau, and V5 positive. The enlarged views were shown in the bottom panel. Scale bars: 100 μm

To further characterize seeding induced tau pathology, control vector (pCMV) or tau-PL-V5 (Tau-PL) transfected cells seeded lysates from control (Ctrl lysate) or rTg4510 (Tg lysate) brains were stained with the PHF1 antibody after 1 % Triton X-100 wash to remove the soluble tau (Fig. 2a). Consistent with the biochemical results, vector transfected cells seeded with Tg lysate (pCMV + Tg lysate) or tau-PL-V5 transfected cells seeded with control lysate (Tau-PL + Ctrl lysate) did not result in detectable PHF1-positive fluorescence. However, 15–20 % of the Tau-PL transfected cells seeded with Tg lysate displayed PHF1-positive staining (Fig. 2a, Tau-PL + Tg lysate). Insoluble tau in cells exhibited cytosolic localization (see Fig. 2a, PHF1 and DAPI Merge). The tau species were also thioflavin-S positive (Fig. 2b), suggesting that seeding induced insoluble tau resembles the neurofibrillary tangles in vivo. Thioflavin-S, total-tau, and V5 staining were almost entirely overlapping, demonstrating that the insoluble tau was converted from cellular soluble tau. Based on the biochemical and immunofluorescent results, we conclude that extracellular seeding can induce intracellular soluble tau to convert to an insoluble and aggregated form reminiscent to the pathological hallmarks of neurofibrillary tangles in aged tauopathy mouse models and human patients.

### Genetic and pharmacological validation of seeding based cell assay

**Fig. 3:**
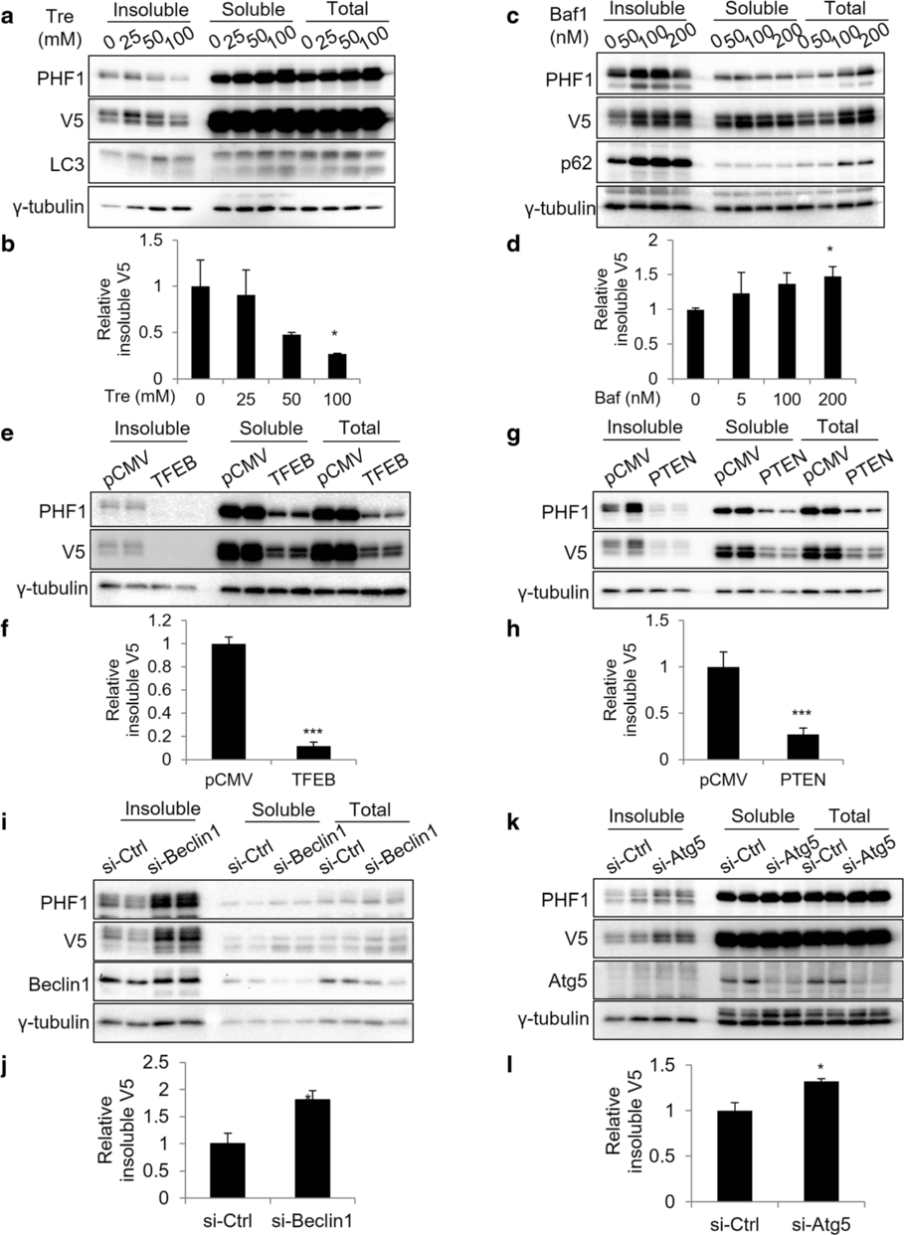
Validation of seeding based cell assay under different conditions. **a** Western blots show decreased insoluble tau in cells treated with different concentrations of trehalose. **b** Quantitative analyses of (**a**). **c** Western blots show increased insoluble tau in cells treated with different concentrations of bafilomycin A1. **d** Quantitative analyses of (**c**). **e** Western blots show decreased insoluble tau in cells transfected with TFEB overexpression plasmid. **f** Quantitative analyses of (**e**). **g** Western blots show decreased insoluble tau in cells transfected with PTEN overexpression plasmid. **h** Quantitative analyses of (**g**). **i** Western blots show increased insoluble tau in cells transfected with Beclin1 siRNA. **j** Quantitative analyses of (**i**). **k** Western blots show increased insoluble tau in cells transfected with Atg5 siRNA. **l** Quantitative analyses of (**k**). Graphical data are expressed as mean ± SEM (***P < 0.001, **P < 0.01, *P < 0.05, *t*-test)

Autophagy is a conserved cellular clearance mechanism, which has been widely reported to degrade pathological tau in vivo [18, 19]. To validate that our seeding based cell assay can simulate the in vivo conditions and is compatible with its regulatory mechanisms, different methods were used to manipulate autophagy in the seeding based cell assay (Fig. 3). Trehalose is a well-documented autophagy activator [20]. In vivo study has demonstrated that trehalose treatment specifically reduces insoluble tau and reverses neurodegeneration [19]. Indeed, trehalose treatment led to dose-dependent increase of soluble and total LC3-II levels (Fig. 3a, LC3 lower band) and corresponding reduction in the insoluble tau (Fig. 3a, Insoluble PHF1 and V5). This was further confirmed by quantification of insoluble V5 tau levels (Fig. 3b). On the contrary, treatment with bafilomycin A1, an autophagy and lysosomal degradation inhibitor, significantly increased p62, a protein marker for autophagic flux (Fig. 3c) [21]. As expected, inhibition of autophagy and lysosomal degradation led to a significant increase in the accumulation of insoluble PHF1 and V5-positive tau (Fig. 3c and quantified in 3d).

We reported previously that transcription factor EB (TFEB), a master regulator of the autophagy and lysosomal pathway, and its downstream target phosphatase and tensin homolog (PTEN), can induce robust tau degradation [22]. Here, we validated that overexpression of TFEB (Fig. 3e and quantified in 3f) and PTEN (Fig. 3g, h) significantly decreased both insoluble and soluble tau in our seeding based assay. Next, we blocked autophagy using siRNA targeting of autophagy-related genes Beclin1 (Fig. 3i, j) and Atg5 (Fig. 3k, l) [23, 24]. Our results showed that both Beclin1 and Atg5 were downregulated by si-RNA knockdown (Soluble and Total, Fig. 3i, si-Ctrl vs. si-Beclin1; Fig. 3k, si-Ctrl vs. si-Atg5). Under these autophagy blockage conditions, insoluble tau was increased as expected (Fig. 3i and k, insoluble PHF1 and V5, with V5 quantified in Figs. 3j and l). This data demonstrate that the seeding based assay is compatible with the three types of most commonly used experimental manipulations: small molecule-based treatment, plasmid mediated gene overexpression, and si-RNA mediated gene knockdown, providing further support for the utility of our seeding based assay for studies of tauopathy.

### Establishment of an image based screening platform

**Fig. 4:**
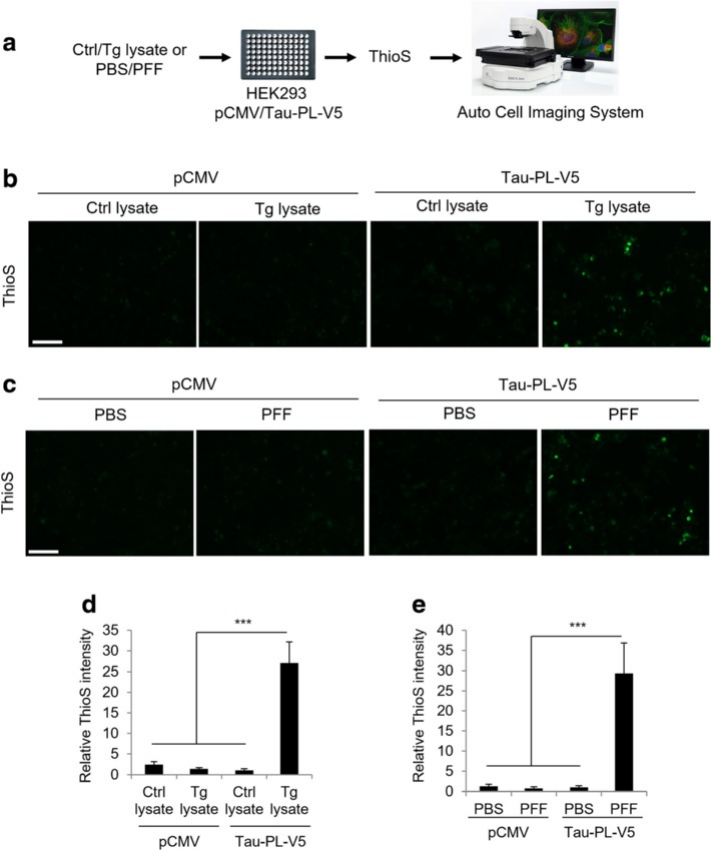
Establishment of image based screening platform. **a** Experimental scheme for the image based screening platform. Seeding assay was performed in 96-well plate and subjected to thioflavin-S staining. Images were captured by EVOS fluorescent microscopy. **b** Representative images show thioflavin-S positive signal only in cells expressing Tau-PL-V5 and treated with Tg brain lysate, but not in other groups. **c** Representative images show thioflavin-S positive staining signal only in cells expressing Tau-PL-V5 and treated with PFFs, but not in other groups. **d** Quantitative analyses of (**b**). **e** Quantitative analyses of (**c**). Graphical data are expressed as mean ± SEM (***P < 0.001, *t*-test). Scale bars: 100 μm

Without a useful cellular model, potential target genes or drugs could only be identified and validated through time and labor intensive animal experiments in tauopathy studies. Here, we retrofitted our seeding based cellular assay to a high content screening modality (Fig. 4a and described in details in Methods). We plated the HEK293 cells expressing the vector or tau-PL-V5 into 96-well plates. After seeding with control or Tg lysate or tau PFF, thioflavin-S staining was performed to determine the amount of cytosolic insoluble tau species. As previously demonstrated, the thioflavin-S positive signal was only detected in cells expressing tau-PL-V5 and treated with Tg lysate (Tau-PL-V5, Tg lysate, Fig. 4b with quantification in 4d) or tau PFF (Tau-PL-V5, PFF, Fig. 4c and quantified in 4e), but not in control lysate treated or vector expressing cells.

### Validation of image based screening platform

**Fig. 5:**
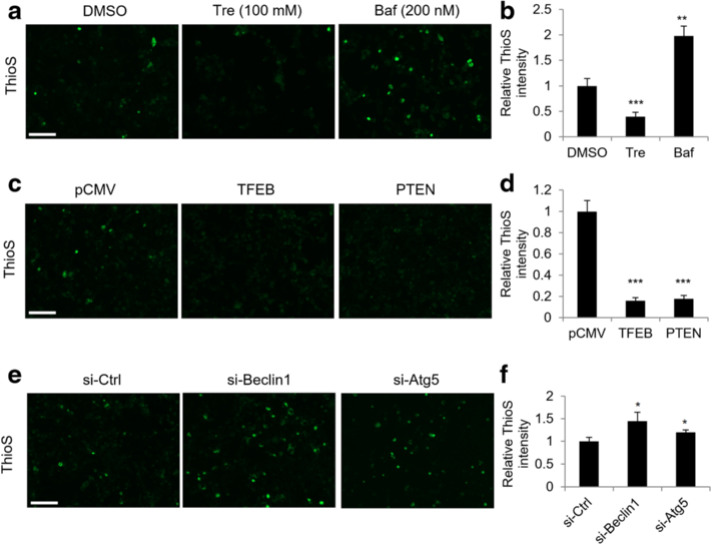
Validation of image based screening platform. **a** Representative images show decreased thioflavin-S signal in cells treated with trehalose and increased thioflavin-S signal in cells treated with bafilomycin A1. **b** Quantitative analyses of (**a**). **c** Representative images show decreased thioflavin-S signal in cells overexpressing TFEB or PTEN. **d** Quantitative analyses of (**c**). **e** Representative images show increased thioflavin-S signal in cells with knockdown of Beclin1 or Atg5. **f** Quantitative analyses of (**e**). Graphical data are expressed as mean ± SEM (***P < 0.001, **P < 0.01, *P < 0.05, *t*-test). Scale bars: 100 μm

To further validate our screening platform, we tested the fluorescence intensity of thioflavin-S staining under various treatment conditions. Consistent with the biochemical results, trehalose (Tre) or bafilomycin A1 (Baf) treatment led to reduced and increased thioflavin-S signal respectively (Fig. 5a and quantified in Fig. 5b). Overexpression of TFEB or PTEN induced significant reduction of fluorescence intensity (Fig. 5c and quantified in 5d). Blockage of autophagy through si-RNA meditated knockdown of Beclin1 or Atg5 resulted in increased fluorescence intensity (Fig. 5e and quantified in Fig. 5f). These data demonstrate that our seeding based cell assay is compatible with different experimental conditions and could be used as a screening system. Autophagy deficit has been implicated in several neurodegenerative diseases including tauopathy. Manipulation of this pathway is considered a potential therapeutic approach for these conditions. Here we validated several autophagy related molecules in our seeding based cell model, including key transcriptional and post-translational regulators of autophagy and small molecule autophagy modulators. Thus this screening system can be used to discover novel pathways or small molecule tau/NFTs modulators for mechanistic studies and therapeutic targeting respectively.

## Conclusion

In this study, we describe a seeding based cellular model of tauopathy based on the prion hypothesis. We demonstrate that extracellular seeding induces the conversion of cellular soluble tau to insoluble thioflavin-S positive tau resembling the classic hallmarks of neurofibrillary tangles in tauopathy mice and human patients. The seeds may be derived from different origins, either aged tauopathy mouse brain lysate or synthetic tau fibers, which allows for flexibility in the application of this assay. We manipulated autophagy via three different techniques: small molecule-based treatment, gene overexpression, and gene knockdown, and validated that upregulation of autophagy decreased insoluble tau, while downregulation of autophagy increased insoluble tau. Thus this assay can be used for investigating tau pathology in vitro. Further, we used thioflavin-S fluorescence to visualize insoluble tau, making the assay compatible with high-content image based screening. In conclusion, our seeding based cellular assay can be easily performed, are subject to various experimental manipulations, and the results are consistent and quantifiable. This assay may be used in pre-validation experiments prior to commencing animal studies of candidate genes or drugs and also offer a possible platform for discovery studies and chemical screening of tauopathy.

## Methods

### Animals

The rTg4510 tau transgenic mice was obtained from the Jackson laboratory and produced by crossing the transactivator line CaMKIIα (129S6 background) with the tau responder line (FVB background) [25]. The wild-type littermates were used as controls. All protocols involving mice were approved by the Institutional Animal Care and Use Committee of Baylor College of Medicine.

### Seeds preparation

For brain lysate, forebrain tissues were lysed in 10 % weight/volume in RIPA buffer (TBS with 1 % Triton X-100, 0.1 % sodium dodecylsulfate and protease phosphatase inhibitor cocktails). Cell lysate was sonicated for ten pulses at output 5, 50 % duty cycle and centrifuged at 12,000 rpm for 15 min. Supernatants were aliquoted and stored in − 80 °C until use. Recombinant tau was purified and fibrillized in vitro to generate PFF as described previously [26]. Briefly, truncated tau containing only four MT-binding repeats (4R) with a Myc tag at the 5’ end and the P301L mutation (5’Myc-K18/P301L) were cloned into NdeI/EcoRI sites in pRK172 bacterial expression vector (generous gift from Virginia Lee). The protein was expressed in BL21 (DE3) RIL cells and purified by cationic exchange using a fast protein liquid chromatography (FPLC). The purified protein underwent in vitro fibrillization by mixing 40 μM recombinant tau with 40 μM heparin and 2 mM DTT in 100 mM sodium acetate buffer (pH 7.0). The protein was then incubated 2–3 days at 37 °C without agitation. Fibrillization was confirmed using the thioflavin T fluorescence assay, sedimentation test, and cryo electron microscopy. Prior to use or storage, the fibrillization mixture was centrifuged at 100,000 × g for 30 min at 4 °C. The resulting pellet was resuspended in 100 mM sodium acetate buffer (pH 7.0), without heparin and DTT, to make a 40 μM solution. The PFFs were then frozen as single use aliquots at − 80 °C. Prior to use in experiments, frozen aliquots were thawed followed by sonication of 30 brief pulses.

### Cell culture, transfection and seeding assay

HEK293 cells were maintained in DMEM (Life Technologies) with 10 % FBS at 37 °C with 5 % CO_2_. Cells were cultured in 60 mm^2^ dish with 5 mL medium. Transfection was performed based on manufacture’s protocol. Tau P301L-V5 was a gift from Dr. Leonard Petrucelli (Mayo Clinic, Jacksonville, Florida, USA), encoding full length human tau with P301L mutation and V5 tag (GKPIPNPLLGLDST). In brief, 2–4 μg tau P301L or pCMV plasmids were diluted with Opti-MEM, then added to lipofectamine 3000 regent and P3000 reagent. After 10–15 mins incubation, DNA-lipid complex was added to medium. One day after transfection, 5 μL brain lysate or 20 μL PFF was added into culture medium. One day after seeding, cells were collected in ice-cold TBS.

### Protein fractionation and western blotting

In one 60 mm^2^ dish, cells were lysed in 1 mL TBS with 1 % Triton X-100 and protease phosphatase inhibitor cocktails. Then the lysate was sonicated with ten pulses at output 4, 50 % duty cycle. 20 μL of supernatant was collected as total protein fraction. The rest was subjected to ultracentrifugation at 100,000 x *g* for 30 min. The 950 μL supernatant was collected as the Triton X-100 soluble fraction. Pellets were washed by 1 mL TBS with 1 % Triton X-100 and subjected to ultracentrifugation at 100,000 x *g* for 30 min again. Pellets from the second ultracentrifugation step were resuspended in 200 μL TBS with 1 % SDS and sonicated with 5 pulses at output 5, 50 % duty cycle, which 200 μL protein buffer was labeled as the Triton X-100 insoluble fraction. All three fractions were boiled in 5XSDS-sample loading buffer. 20 μL of each fraction was loaded per gel for SDS-PAGE followed by western blot.

### Immunofluorescence assay

For immunostaining of insoluble tau, cells grown on cover slips were fixed with 4 % paraformaldehyde and 1 % Triton X-100 at room temperature for 15 min. Cells were incubated overnight with primary antibodies in TBS blocking solution containing 0.4 % Triton X-100. After washing, cells were incubated for 2 h with secondary antibody. For thioflavin-S staining, cells were incubated with 0.002 % thioflavin-S in TBS, followed by rinsing with 50 % EtOH twice, then 5 min in TBS before imaging. Images were captured by EVOS fluorescent microscopy.

## Abbreviations

AD: Alzheimer’s disease
Baf: bafilomycin A1
NFTs: neurofibrillary tangles
PFF: preformed tau fibrils
PTEN: phosphatase and tensin homolog
RD: repeat domain
TFEB: transcription factor EB
ThioS: thioflavin-S
Tre: trehalose
